# Mentorship in nursing academia: a systematic review protocol

**DOI:** 10.1186/s13643-015-0007-5

**Published:** 2015-02-21

**Authors:** Lorelli Nowell, Deborah E White, Kelly Mrklas, Jill M Norris

**Affiliations:** Faculty of Nursing, University of Calgary, 2500 University Drive NW, Calgary, AB T2N 1N4 Canada; Department of Community Health Sciences, Cumming School of Medicine, University of Calgary, 3280 Hospital Drive NW, Calgary, AB T2N 4Z6 Canada

**Keywords:** Mentorship, Mentors, Career choice, Career mobility, Nursing, Nurses, Academia, Faculty, Systematic review

## Abstract

**Background:**

Mentorship is perceived as vital to attracting, training, and retaining nursing faculty members and to maintaining high-quality education programs. While there is emerging evidence to support the value of mentorship in academic medicine, the extant state of the evidence for mentorship in nursing academia has not been established. We describe a protocol for a mixed-methods systematic review to critically appraise the evidence for mentorship in nursing academia.

**Methods:**

Studies examining the effectiveness of mentorship interventions with nursing faculty who teach in registered nursing education programs will be included. Mentee, mentor, and nursing education institutional outcomes will be explored. Quantitative, qualitative, and mixed method studies will be eligible for inclusion, without restrictions on publication status, year of publication, or language. We will search electronic databases (for example, MEDLINE, CINAHL, ERIC) and gray literature (for example, conference proceedings, key journals, relevant organizational websites) for relevant citations. Using pilot-tested screening and data extraction forms, two reviewers will independently review the studies in three steps: (1) abstract/title screening, (2) full-text screening of accepted studies, and (3) data extraction of accepted studies. Studies will be aggregated for meta-synthesis (qualitative) and meta-analysis (quantitative), should the data permit.

**Discussion:**

This study is the first systematic review of existing global evidence for mentorship in nursing academia. It will help identify key evidence gaps and inform the development and implementation of mentorship interventions. The mentorship outcomes that result from this review could be used to guide the practice of mentorship to increase positive outcomes for nursing faculty and the students they teach and ultimately effect improvements for the patients they care for. This review will also identify key considerations for future research on mentorship in nursing academia and the enhancement of nursing science.

## Background

One of the many challenges in nursing education today is the shortage of nursing faculty [1]. In a report focusing on human resources for health, the World Health Organization described a shortage of nurse faculty in the majority of its member states in 2006 [2]. The number of nurses in the workforce continues to decrease, as does the number of nursing faculty needed to teach new nurses to ensure quality health care delivery, to study health problems, to address patient issues, and to inform health policy. Nursing faculty shortages have not received the same attention as registered nursing (RN) shortages, but the problem is no less critical. The shortage of qualified nursing faculty is an issue of local, national, and international concern and is anticipated to worsen [3].

Nurses are the largest health-care professional group, comprising approximately 51% of all health-care providers globally, ranging from the lowest (47%) in Europe to the highest (71%) in Southeast Asia [2]. Diminished nursing faculty directly impacts the ability to admit and graduate adequate numbers of students for the nursing workforce [3-5], which further impedes resolution of workforce shortages. This is of concern because nurses spend more direct time with patients than any other health-care professionals and play a critical role in health outcomes [6-8]. The shortage of qualified RNs has been shown to decrease quality of health-care delivery [6-9]. Growing nurse faculty shortages are a threat to patient outcomes [10,11].

The nursing faculty shortage has implications for nursing research and its influence, particularly at a time when health system transformation is of paramount importance globally [12]. Generation, dissemination, and application of evidence is essential to maintain and expand any discipline [13], and the recognition of nursing as a profession and academic discipline is greatly dependent on evidence-based practice, with nursing knowledge imparted through education and advanced through scholarship [14]. According to Wood *et al*. [15], as energy is focused on stemming the shortage of nurses for the health-care system, the capability to build critical research capacity may be lost. Deliberate attention must be given to scholarship in order for the nursing discipline to advance and keep pace with parallel advancements in medical and related subspecialties, to advance evidence-based practice, and to inform effective, sustainable health care. The absence of an academic nursing presence from front-line care, administration, research, and policy arenas is of long-term detriment to patient outcomes and the nursing profession. The expansion of nursing science has shown to be instrumental in the provision of better patient care and improved health [16]. There are not currently enough master’s- and doctorally trained nurses to meet increasing research and leadership demand [16,11].

In 2004, Wood, Giovanetti, and Ross-Kerr [15] acknowledged that the number of doctoral students would not sufficiently meet the needs of nursing schools across Canada. Five years later, the Canadian Nurses Association (CNA) and Canadian Association of Schools of Nursing (CASN) reported a need for 3,673 nurses with master’s degrees and 650 nurses with doctoral degrees to meet existing school of nursing faculty vacancies [11]. The CNA and CASN continue to warn of an imminent shortage of qualified faculty if current entry-to-practice enrolments are maintained [4]. Diminished enrollees and graduates in doctoral nursing programs have also been acknowledged in the USA. In 2004, the American Association of Colleges of Nursing (ACCN) reported that insufficient number of faculty resulted in over 75,000 qualified applicants being refused entrance into baccalaureate, master’s, and doctoral nursing programs [17]. Although insufficient statistics are currently available from Australia, New Zealand, and the United Kingdom, the Council of Deans of Nursing and Midwifery of Australia and New Zealand have warned that an academic nursing workforce shortage is imminent [18].

The shortage of graduate students, faculty, and researchers persists in the nursing discipline. There is an urgent need to advance evidence-based nursing practice and skills focused on increasing population health, more efficient and effective health services and systems, and returning value on public investments. Nursing faculty shortage will hinder the ability to educate future nurses, erode the potential for research to advance clinical practice, and result in the loss of nursing leadership needed to advance the profession [19].

### Causes of the nursing faculty shortage

According to IOM [16], the root causes of this inability to meet undergraduate nursing educational needs were partly due to nursing faculty shortages, inadequate workforce planning, and decreasing educational capacity to meet market demand. The following key influences have been cited: (a) salary disparities, (b) aging academic workforce, (c) changing faculty workload demands and role expectations, (d) career opportunities in clinical and private sectors, (e) diminished student numbers preparing for faculty positions, and (f) inadequate institutional funding for additional faculty positions.

Nursing faculty are one of the most poorly compensated categories in the nursing profession [16]. According to Kowalski and Kelley [20], equivalent clinical careers paid 25 to 50% more than academic careers, with the cost of acquiring faculty degrees increasingly prohibitive. Large discrepancies between faculty and non-academic salaries persist and negatively impact enrolment and retention [21].

While nursing faculty members are within the same demographic era that has partly influenced the current lack of RNs, academic nursing is further impacted by more rapid aging out of employment than clinical nursing due to later career stream entry [22]. Nursing faculty tend to be older than clinical nurses given advanced degree requirements to teach [16]. This does not lend itself to lengthy employment in an academic setting. Resignation and retirements are projected to reduce the current nursing faculty greatly over the next decade [14]. As aging faculty contributes to attrition, it is important that the next generation of nursing faculty be identified early, encouraged, nurtured, and welcomed into academia [23]. Faculty mentorship is suggested as a way to successfully foster a collegial, caring environment; these supportive relationships are positive strategies that help to retain RNs in faculty positions [24].

The number of master’s- and doctoral-prepared advanced practice nurses choosing academia has decreased throughout the years [21]. Recent statistics indicate that nurses graduating from master’s and doctoral programs are not choosing an academic career path [25]. Increased opportunities outside academia for PhD-trained nurses further contribute to the shortage of nursing faculty [26].

Even if adequate enrolment were not a problem, both US and Canadian nursing programs have lacked the funds to create new teaching positions [22]. Canadian nursing schools have identified the lack of sustainable funding to create full-time positions as a major challenge, limiting their ability to recruit new faculty [4].

Nursing faculty shortage is the result of multiple, systemic problems emerging over decades. With such staggering nursing faculty workforce statistics, recruitment and retention of new nursing faculty are critical strategies. Mentorship has been identified by the National League for Nursing [27] as one way to address the nursing faculty shortage by encouraging RNs to begin and remain in nursing faculty roles.

### Evidence for mentorship

The evidence base for mentorship interventions has evolved in business, medicine, and education literature. Research on mentorship in nursing is a recent development. Most mentorship studies conducted to date are observational or qualitative, and the conclusions are not conclusive. Systematic reviews on mentorship in corporate settings have reported increased job satisfaction and perceived increases in career advancement opportunities for those that received mentorship, compared to those who did not [28]. A systematic review of mentorship in academic medicine reported that mentorship has a significant influence on personal development, career guidance, career choice, and research productivity, recruitment, and retention [29]. Within the education literature, similar reviews have identified mentorship as improving the socialization, orientation, and career outcomes of faculty [30]. Evidence of mentorship in nursing academia has not yet been synthesized.

Nursing education institutions that have established mentoring programs reported positive outcomes for nursing faculty such as improved morale, higher career satisfaction, increased self-confidence, increased professional development, increased publication, obtaining more grants, and quicker promotion [31,32]. Organizations have reported benefits from mentoring including developing future leaders from within the institution through nurturing commitment, retention, and teamwork [33,34]. While the nursing literature contains numerous references to the importance of mentoring, mentorship in nursing academia is not an established standard practice.

Given the potential importance of mentoring in nursing academia, a systematic review is needed to identify and describe how mentoring interventions in the field of nursing academia were developed, implemented, and evaluated. These data will help determine whether there is a sufficient range of methodologically rigorous evidence to support the development of mentorship interventions in nursing academia. This systematic review may also contribute a gap analysis and guide the objectives and designs of future mentorship interventions in nursing academia.

### Aim

The systematic review question is: What is the nature and strength of the evidence for mentorship in nursing academia? The main objective of this mixed-methods systematic review is to evaluate the quantitative and qualitative literature that addresses mentorship in nursing academia. Findings that are relevant to the mentee, mentor, and nursing education institution will be included. Findings that address outcomes, including but not limited to knowledge, skills, attitudes, career progression, recruitment, retention, and costs, will be reported.

## Methods/design

This mixed-methods systematic review protocol is based on the PRISMA [35] and ENTREQ [36] reporting guidelines. The design follows the Joanna Briggs Institute (JBI) [37] approach for conducting systematic reviews of both quantitative and qualitative research. The synthesis of quantitative and the qualitative evidence will be conducted independently prior to a final mixed methods synthesis (that is, segregated). The findings will be presented in a way that preserves the context of their production by anchoring the findings to sample information, source of information, information about time, comparative reference points, information about the magnitudes and significance, and study-specific conceptions of phenomena [38]. This will be facilitated by JBI-SUMARI software (v 5.0; Joanna Briggs Institute, Adelaide, SA, Australia) and analytical modules, including the Meta-Analysis of Statistics Assessment and Review Instrument (JBI-MAStARI), Qualitative Assessment and Review Instrument (JBI-QARI), and Mixed Methods Assessment and Review Instrument (JBI-MMARI) [39]. This protocol has not been registered with PROSPERO, as protocols for systematic reviews of studies not related to health conditions and health-related outcomes are not currently eligible for registration.

### Eligibility criteria

The question of relevance is: What is the nature and strength of the evidence for mentorship in nursing academia?

#### Participants

Studies will be included if they involve RNs teaching in RN education programs. This will include nursing instructors, nursing faculty, nursing researchers, and nursing academics. Studies involving undergraduate nursing students, staff nurses, nursing educators who teach in licensed nursing programs, and/or nursing assistant programs will be excluded. In studies where it is unclear that participants meet our inclusion criteria, we will contact the corresponding study author for verification. We will exclude studies where verification of inclusion criteria is not possible.

#### Interventions

Studies that explore formal and informal mentorship interventions including, but not limited to, dyadic mentoring, peer mentoring, online mentoring, and tele-mentoring will be included.

#### Outcomes

Informed by other medical, education, and business studies, this review will report on the outcomes of measures that are relevant to the mentee, mentor, and nursing education institutions. Similar to other published non-nursing meta-analyses on mentorship [40,41], variables that are conceptually similar will be combined. Table 1 lists the six broad categories of outcomes that will be examined. Within each category, we list the specific outcomes that will be examined and example of how these outcomes are measured. Some of the outcomes listed are applicable to mentors, mentees, and nursing education institutions. We will include new variables if reported.

**Table 1:** **Outcomes of mentoring**

**Mentorship outcomes**	**What is measured**	**How it is measured**
Behavioral		
Performance	Scholarly productivity (grants, publications)	Review of mentor and mentee CVs
Retention	Numbers of mentees and mentors retained	Departmental annual reports
Recruitment	Numbers of recruits stating mentoring influenced their decision to join the faculty	Surveys of faculty recruited
Attitudinal		
Situational satisfaction	Job satisfaction, organizational commitment	Mentor and mentee surveys and interviews
Career attitudes	Career satisfaction, career expectations, perceived employment opportunity	Mentor and mentee surveys and interviews
Health-related		
Self-perceptions	Self-esteem, self-efficacy	Mentor and mentee surveys and interviews
Relational		
Interpersonal relations	Positive peer relations, satisfaction with coworkers, peer support, relationship quality	Mentor and mentee surveys and interviews
Motivational		
Motivation/involvement	Career planning, job involvement, motivation, aspiration, career commitment	Mentor and mentee surveys and interviews
Career		
Career recognition and success	Academic rank, promotion	Review of mentor and mentee CVs
Skill competence and development	Work knowledge, academic socialization, self-efficacy with academic skills	Departmental annual reports
		Mentor and mentee surveys and interviews

CVs, curriculum vitae.

#### Study type

The review will include quantitative, qualitative, and mixed method studies that report on mentorship on nursing academia without restriction by study design, publication status, year of publication, or language.

### Information sources and search strategy

Prior to commencing the study search, a preliminary search of existing systematic reviews will be made through Database of Abstracts and Reviews (DARE), MEDLINE, and PROSPERO to identify studies relevant to this review. Electronic searches will include MEDLINE, CINAHL, EMBASE, ERIC, and PsycINFO databases from their inception to present, and the search strategy will be updated within 90 days of final publication, without limitation on study design, publication year, status, or language. A search to identify gray literature (non-peer-reviewed works) will be undertaken by scanning ProQuest Dissertations and Theses, Index to Theses, and mentorship conference proceedings. Experts in the field and corresponding authors of key studies will be contacted to gather further information on gray literature. The authors will undertake a bibliographic search of all eligible studies to identify and retrieve other relevant studies for the review.

The search strategy was designed with the assistance of an experienced nursing librarian to focus on maximum sensitivity and to be as extensive as possible to identify all possible eligible studies and then refined according to the inclusion and exclusion criteria. Several consecutive searches were performed and the results were combined to design the final search strategy. The provisional search strategy for MEDLINE is outlined in Table 2 and will be modified according to the indexing systems of the other databases. All references will be exported to EndNote citation management software, where duplicated records will be verified, recorded, and removed.

**Table 2:** **Provisional search strategy for Ovid MEDLINE**

	**Search strategy**
1.	exp *Mentors/
2.	mentor*.mp.
3.	1 or 2
4.	exp*Nursing/
5.	nurs*.mp.
6.	4 or 5
7.	exp *Nursing Faculty Practice/or exp *Faculty, Nursing/ or faculty.mp.
8.	exp *Universities/ or academia.mp.
9.	instructor*.mp.
10.	university.mp
11.	college.mp
12.	academic.mp
13.	educator.mp
14.	7 or 8 or 9 or 10 or 11 or 12 or 13
15.	3 and 6 and 14
16.	Remove duplicates from 15

### Study selection

The selection of studies will occur in two phases. The first phase will involve screening of titles and abstracts by two reviewers, independently using a structured data entry form. To minimize the risk of bias, data screening forms will be pilot tested by reviewers on the first 50 studies to ensure consistency and reliability. A Kappa [42] of greater than 0.6 will be used to quantify inter-investigator agreement. Disagreements will be resolved to consensus through discussion and passed to a third investigator for final resolution if the issue cannot be resolved. Studies identified as potentially relevant will be passed to the next screening level.

In phase two, the same two reviewers will independently review full-text versions of all potentially relevant studies. To minimize the risk of bias, both reviewers will be trained on the use of the eligibility form prior to beginning the review. Eligibility forms will be pilot tested by the reviewers on the first ten identified full texts to ensure consistency and reliability between the reviewers. A Kappa [42] of greater than 0.6 will be used to quantify inter-investigator agreement, and disagreements will be resolved by discussion. Unresolved disagreements will be referred to a third investigator for review and resolution.

### Data collection process and data items

Once a final set of included studies is established, data will be extracted independently by two researchers according to the inclusion and exclusion criteria using two standardized data extraction instruments: one specific to quantitative studies (JBI-MAStARI) and one specific to qualitative studies (JBI-QARI). To minimize the risk of bias, reviewers will be trained on both data extraction forms prior to extracting data. The data extraction forms will be pilot tested by the reviewers on the first ten included studies to ensure consistency and reliability between the reviewers. Disagreements will be resolved by discussion. In the absence of consensus, disagreements will be referred to a third investigator for review and resolution. Table 3 shows data categories that will be extracted from all the studies selected.

**Table 3:** **Data categories extracted from included studies**

**Data category**	**Data extracted**
General information	ID numbers, authors, title of article, type of publication, year of publication, and language
Study characteristics	Aim of the study, study designs, inclusions and exclusion criteria, recruitment procedures and sample size
Participant characteristics	Age (mean/SD), gender, years of experience as a RN (mean/SD), years of experience in academia (mean/SD), and other characteristics of interest described by the authors of the studies
Setting	Country, institution, and other setting characteristics described by the authors of the studies
Mentorship interventions	Description of the mentorship intervention(s) and how the intervention(s) was developed, implemented, and evaluated
Outcomes	Primary and secondary outcomes and definition for each outcome reported
Ethical characteristics	Ethics approval, informed consent, information provided to participants

RN, registered nurse; SD, standard deviation.

Studies that have been published in duplicate will be retained and assessed in full text; the most comprehensive study will be included. Following independent data extraction, co-reviewers will meet to resolve any discrepancies and obtain consensus. Any unresolved disagreement between the two reviewers will be solved by referral to a third researcher.

### Assessment of methodological quality/risk of bias in individual studies

Each included study will be assessed for methodological quality by two independent reviewers. Quantitative studies will be assessed using the appropriate JBI-MAStARI critical appraisal tool for controlled trial/pseudo-randomized trial, comparable cohort/case control studies, or descriptive/case series studies. All qualitative studies, regardless of study design, will be assessed using JBI-QARI critical appraisal tool. Responses to these quality appraisal questions are:

- ‘Yes’ (the criteria have been established through the report description or have been confirmed by the primary author)
- ‘No’ (the criteria have not been applied appropriately)
- ‘Unclear’ (the criteria are not clearly identified in the report and it was not possible to acquire clarification from the author)
- ‘Not Applicable’

When both reviewers have completed the assessment process, the primary reviewer will compare the two sets of appraisals. Any discordant response will be first discussed by the first two reviewers and referred to a third reviewer if a resolution cannot be reached. All non-English literature identified in the search will be screened and reviewed by one interpreter. Studies that meet the inclusion criteria will be extracted by the same interpreter.

### Synthesis of included studies

Mentorship studies are known to be heterogeneous; if possible, the quantitative data will be pooled for meta-analysis using JBI-MAStARI and we will use a random-effects model (odds ratios for categorical data, mean differences for continuous data, 95% confidence intervals). Meta-aggregation will be used to synthesize qualitative date using JBI-QARI, if possible. This process will involve assembling the findings based on study quality, categorizing findings based on similar meanings, and producing a set of synthesized findings. If there is a lack of available studies and statistical or textual pooling is not achievable in the single method syntheses, findings will be reported in narrative form. JBI-MMARI will be used to aggregate the single-method syntheses. Using a Bayesian approach, the quantitative findings will then be converted to qualitative themes and subsequently pooled with the qualitative synthesis in tabular form. This approach was used by Crandell and colleagues [43], whereby similar variables will be grouped together into themes and then data will be coded for each variable. These data will be entered into a data matrix with included studies in rows and single themes (or variables) in the columns. If a study does not address a variable, that cell will be left blank. An overarching synthesis will be created for each theme (based on the variables included in that theme). Utilizing both quantitative and qualitative data to develop themes and coding all data into a compatible format allows for a meta-aggregative analysis where equality between qualitative and quantitative data is achieved. Finally, all themes will be aggregated to generate a set of recommendations for mentorship in practice and mentorship research.

### Ethical considerations

Systematic reviews should not ignore ethical considerations [44]. An ethical assessment will be conducted for all included studies in this systematic review, and an assessment of ethics approval for all gray literature will be confirmed. The ethical characteristics will be collected and summarized in the discussion of the systematic review findings.

### Validity and reliability

In order to ensure decisions are not biased, a systematic review team has been established to conduct this systematic review. The team includes a knowledge expert with a research focus on mentorship, systematic review methodologists, and a nursing research librarian. All team members will participate in regularly scheduled meetings to discuss project progress and findings. To minimize the risk of error, reviewers will be trained on the use of all selection, appraisal, and extraction forms prior to beginning the review. The forms will be pilot tested by the reviewers to ensure consistency and reliability between the reviewers.

## Discussion

This study is the first systematic review of existing global evidence for mentorship in nursing academia. It will help identify key evidence gaps and inform the development and implementation of mentorship interventions. The mentorship outcomes that result from this review could be used to guide the practice of mentorship to increase positive outcomes for nursing faculty and the students they teach and ultimately effect improvements for the patients they care for. This review will also identify key considerations for future research on mentorship in nursing academia and the enhancement of nursing science.

**Figure 1:**
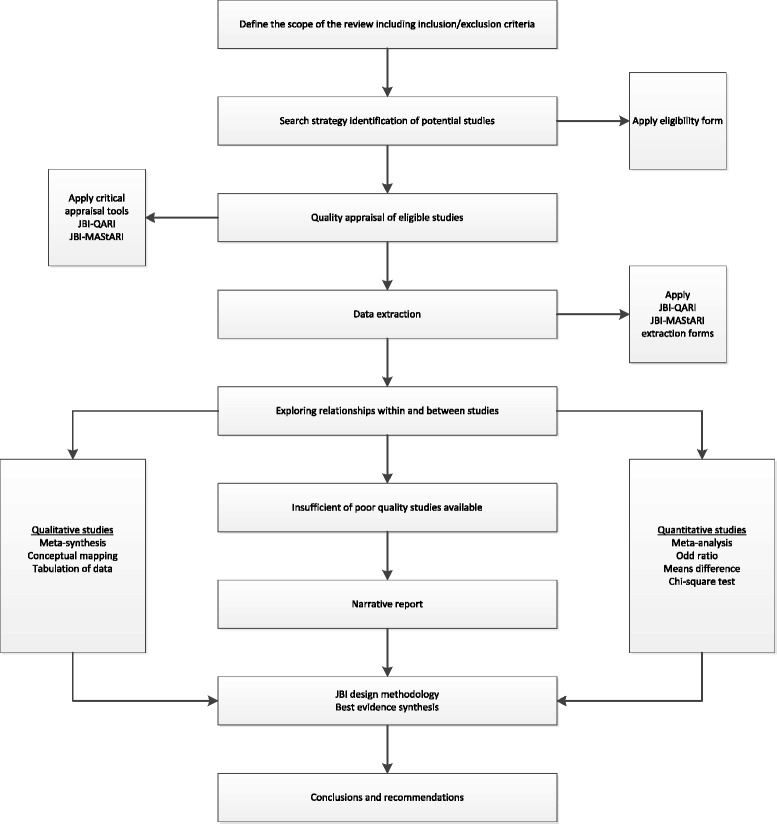
**Systematic review development.** JBI, Joanna Briggs Institute; JBI-MAStARI, Joanna Briggs Institute Meta-Analysis of Statistics Assessment and Review Instrument; JBI-QARI, Joanna Briggs Institute Qualitative Assessment and Review Instrument.

This systematic review protocol considers both quantitative and qualitative studies. Mixed methods reviews are still evolving and consistent methods have not been validated. In response to these concerns, the development process of this systematic review is illustrated in Figure 1. The methodology used has been adapted from JBI [37] and other mixed methods systematic reviews [45]. The robust method of this systematic review protocol enables critical appraisal and synthesis of the cumulate global evidence on the topic, while preserving the integrity of findings from different study designs and providing precise results with rich contextual data.

The current lack of knowledge synthesis is a major limitation of the current state of evidence on mentorship strategies aimed at addressing the nursing faculty shortage. Although a number of mentorship outcomes (increased recruitment, retention, promotion, job satisfaction, occupation commitment, career progression, skills development, self-efficacy, publications, grants, and decreased administrative costs) have been identified in medicine, business, and education literature, presently, the outcomes of mentorship in academic nursing remain unclear. The absence of a systematic review that identifies, critically appraises, and synthesizes the current evidence for mentorship interventions presents a dilemma for policy makers. Failing to provide a consensus understanding of appropriate mentorship approaches and positive mentorship outcomes has left policy makers with limited guidance regarding which alternatives to consider when designing mentorship strategies to alleviate the nursing faculty shortage [46]. We have planned this review to address this current knowledge gap.

The findings of this systematic review may have implications for policy, practice, and research. The results of this systematic review will provide a comprehensive examination of the evidence for mentorship in nursing academia and highlight gaps where future research on mentorship remains to be conducted. Given the significant resources required to fund mentorship innovations, understanding the benefits and shortcomings of various strategies may ensure that scarce resources are devoted to the most efficient and effective strategies. The result from this review could be used to guide administrators and policy makers to most effectively implement mentorship innovations aimed at addressing the nursing faculty shortage.

### Limitations

Due to the complexities and diversity of mentorship interventions and limited availability of quantitative studies, the extent to which clear conclusions can be drawn about the usefulness of mentorship may be limited. However, this review will provide clarity on the existing evidence for mentorship in nursing academia and identify areas for future research.

## Abbreviations

ACCN: American Association of Colleges of Nursing
CNA: Canadian Nurses Association
CASN: Canadian Association of Schools of Nursing
JBI: Joanna Briggs Institute
JBI-MAStARI: Joanna Briggs Institute Meta-Analysis of Statistics Assessment and Review Instrument
JBI-MMARI: Joanna Briggs Institute Mixed Methods Assessment and Review Instrument
JBI-QARI: Joanna Briggs Institute Qualitative Assessment and Review Instrument
JBI-SUMARI: Joanna Briggs Institute System for the Unified Management, Assessment and Review of Information
RN: Registered nurse
